# Behavioral and electrophysiological aspects of cognition in neonate rats lactated by morphine addicted mothers

**DOI:** 10.22038/ijbms.2019.36892.8789

**Published:** 2019-09

**Authors:** Fatemeh Aghighi, Mojgan Mohammadifar, Hamidreza Banafsheh, Mahmoud Salami, Sayyed Alireza Talaei

**Affiliations:** 1 Physiology Research Center, Institute for Basic Sciences, Kashan University of Medical Sciences, Kashan, Iran

**Keywords:** Hippocampus, Lactation, Maze learning, Morphine, Rats, Spatial memory, Synaptic plasticity

## Abstract

**Objective(s):**

In addition to genetic factors, environmental phenomena during postnatal age highly affect development and, in turn, function of the brain. The present work evaluates if morphine consumption during lactation period influences the spatial performances and synaptic plasticity in rats at neonatal period of age.

**Materials and Methods:**

Three groups of mothers were subcutaneously administered by 5 (M5), 10 (M10) or 20 (M20) mg/kg morphine every 12 hours during the lactation period. At 45 days old, their offspring were introduced to Morris water maze for assessment of spatial learning and memory. Basic field excitatory post-synaptic potentials (fEPSPs) were recorded in the CA1 area of hippocampus and, then, long term potentiation (LTP) was induced by tetanic stimulation.

**Results:**

We found that the M10 and M20 rats spent more time and traveled longer distance to find the hidden platform of maze when compared to the control animals (*P*<0.05 for all comparisons). Similarly, these two morphine-exposed groups were inferior in the memory consolidation compared to their control counterparts. Comparing control and M20 rats revealed that morphine exposure decreases the mean amplitude and slope 10-90% of fEPSPs about 30 percent (*P*<0.001 for both comparisons) and inhibits the LTP induction in the CA1 area circuits.

**Conclusion:**

The present study provides behavioral and electrophysiological proofs for negative effect of morphine on the hippocampal-related function in the neonatally morphine-exposed rats.

## Introduction

Although opioids, specifically morphine, are of the most commonly prescribed analgesics, but they are severely addictive, and repeated or chronic use of them has destructive neuro-biological consequences (1). Chronic morphine consumption alters synaptic plasticity of neural circuits of different area of the brain, especially the hippocampus (2). The hippocampus has a crucial role in early stages of learning and memory formation (3). It is not exactly clear how memories form but two types of synaptic plasticity namely long-term potentiation (LTP) and long-term depression (LTD) have been accepted as key mechanisms of memory formation, theoretically (4). In one way, the hippocampus has an essential role in memory formation and in other way, there is a bilateral relationship between this area and the neurocircuitry of addiction, so it is important to evaluate the effects of morphine on synaptic plasticity of hippocampal neural circuits (5). It has been shown that opioid receptors, especially μ opioid receptors (MOR), are distributed in the hippocampus (6). There are several studies suggesting that morphine modulates hippocampus-dependent learning and memory and synaptic plasticity of hippocampal neural circuits in both human and animal models (7). For example, it has been demonstrated that chronic morphine exposure (10 mg/kg daily for 7 days) causes a marked inhibition of LTP in the CA1 area of rat’s hippocampus (8). Conversely, Miladi-Gorji *et al.* have shown that chronic morphine exposure (10 mg/kg twice daily for 10 days) augments LTP in the dentate gyrus of rat hippocampus (9). In addition, results of several studies have confirmed that acute or chronic morphine exposure impairs spatial learning and memory (10) or passive avoidance memory (11). 

 Neural circuits of the mammals’ brain are shaped by exposure to the environment in addition to genetic codes during prenatal and early postnatal period, named critical period of brain development (12). The hippocampus has a critical period of development like the other areas of the brain (13). Many studies have demonstrated that morphine consumption during pregnancy has prolonged effects on structure and function of offspring neural circuits (14, 15). Yang and colleagues have reported that although morphine exposure during pregnancy decreases the magnitude of LTD of hippocampal neural circuits of rats’ offspring, it has no effect on the LTP expression (16). Morphine consumption during pregnancy and lactation causes dependency of 14 days old rat offspring and reduces antinociceptive effect of morphine (17). In addition, it has been shown that morphine consumption during pregnancy and lactation decreases N-methyl-D-aspartate (NMDA) receptors (18), and phosphorylated CREB (19) in the brain of 14 days old rat offspring, and changes the kinetics of hippocampal NMDA receptors (20). The aim of this study is evaluating the effects of morphine consumption during lactation period on spatial learning and memory and synaptic plasticity of hippocampal neural circuits of rats’ offspring.

## Materials and Methods

***Animals***

This experimental study was carried out on 40 male Wistar rats (45 days of old, weighting 120-150 g). The rats were housed in standard animal house at 12/12 hr light-dark cycle, temperature of 22±2°C and air humidity of 55±5% with food and water *ad libitum*. All experiments were approved by Ethical Committee of Kashan University of Medical Sciences, Kashan, Iran and were carried out in accordance with Directive 2010/63/EU on the protection of animals used for scientiﬁc purposes. The rats were allocated into the following groups (n=10 for each): Control animals (CO) and the rats that their mothers received 5 (M5), 10 (M10) or 20 mg/kg (M20) morphine sulfate (Temad, Iran) during lactation period every 12 hr, subcutaneously. It should be mentioned that only two offspring were selected from each morphine-received mother. All the offspring were weaned at postnatal day 21, two hours after receiving the last dose of morphine by their mothers and the withdrawal symptoms were checked as follows: Naloxane hydrochloride (Toliddarou, Iran) was administered at dose of 2 mg/kg intraperitoneally. Immediately after the injection, the animals were placed in a plexiglass chamber (30×30×50 cm) and their behavior signs were observed for 30 min according to a modiﬁed version of the Gellet–Holtzman scale (21). Briefly, these signs include graded (body weight loss during 24 hr after the Naloxane injection, jumps, abdominal contractions, and wet dog shakes) and checked signs (irritability, writhing, diarrhea, ptosis, erection or genital grooming, and teeth chattering). 

**Figure 1:**
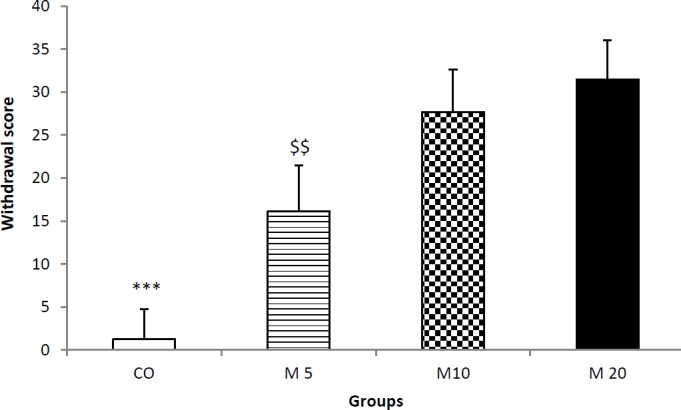
The effect of receiving morphine during lactation period on dependency of rats’ offspring. Results showed that as the morphine dose increased, the overall Gellert-Holtzman score increased. Data are shown as means±SEM. *** *P*<0.001 control group vs. the other groups

$$ *P*<0.01 M5 group vs. M10 and M20 groups

**Figure 2:**
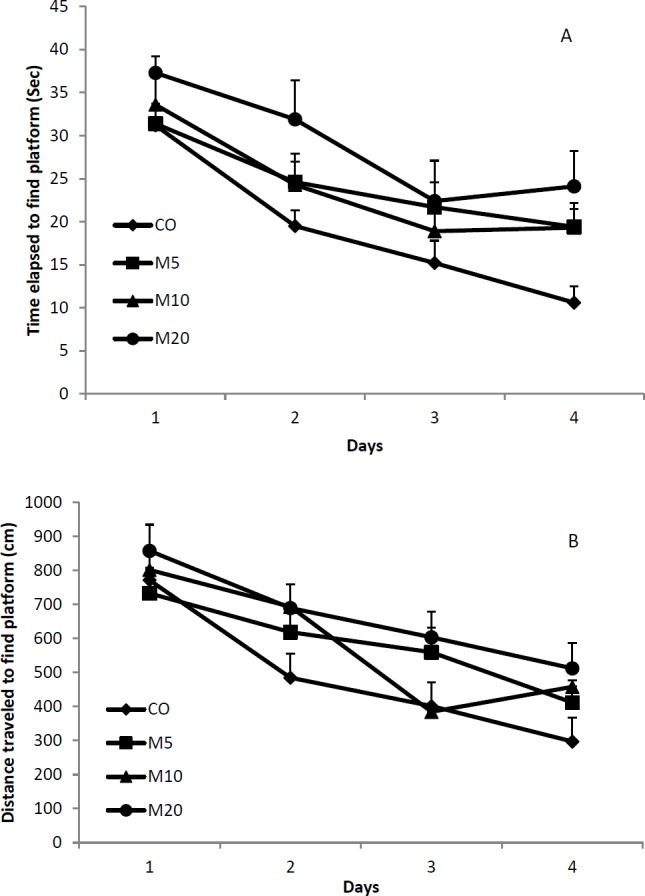
Performance of the different groups of rats in the Morris water maze is shown as the required time (A) and the traveled distance (B) to find the hidden platform. Receiving 10 or 20 mg/kg morphine twice daily during lactation period causes that the rats’ offspring spend more time (*P*<0.05 for both comparisons) and travel more distance (*P*<0.05 for both comparisons) to find the hidden platform of Morris water maze than the control rats. Data are shown as means±SEM and each point indicates the average of four daily trials

**Figure 3:**
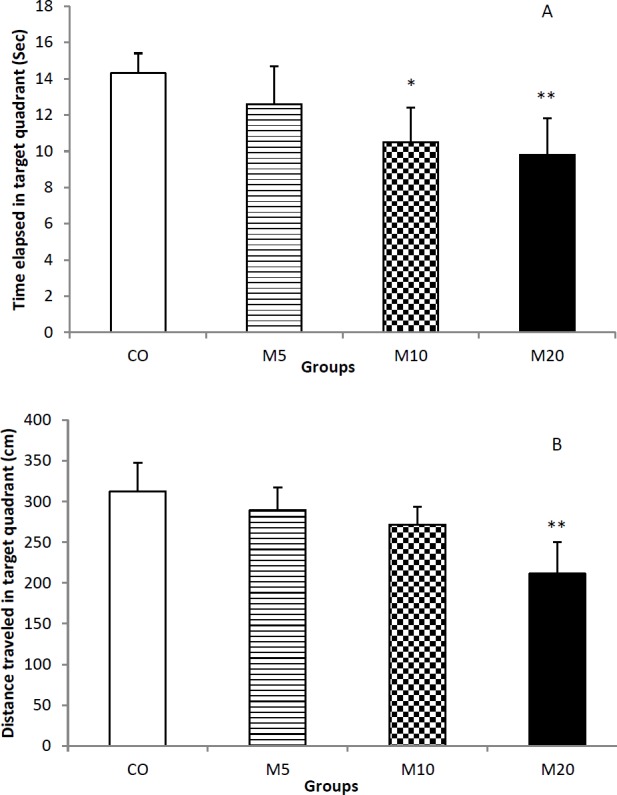
Spatial memory was evaluated at the fourth day in the probe trial as mean spent time (A) and mean traveled path (B) in the target quadrant. Morphine consumption during lactation period causes that the rats’ offspring spend less time and travel less distance in the target quadrant, dose-dependently.* *P*<0.05 control group vs. M10 group; ** *P*<0.01 control group vs. M20 group

**Figure 4:**
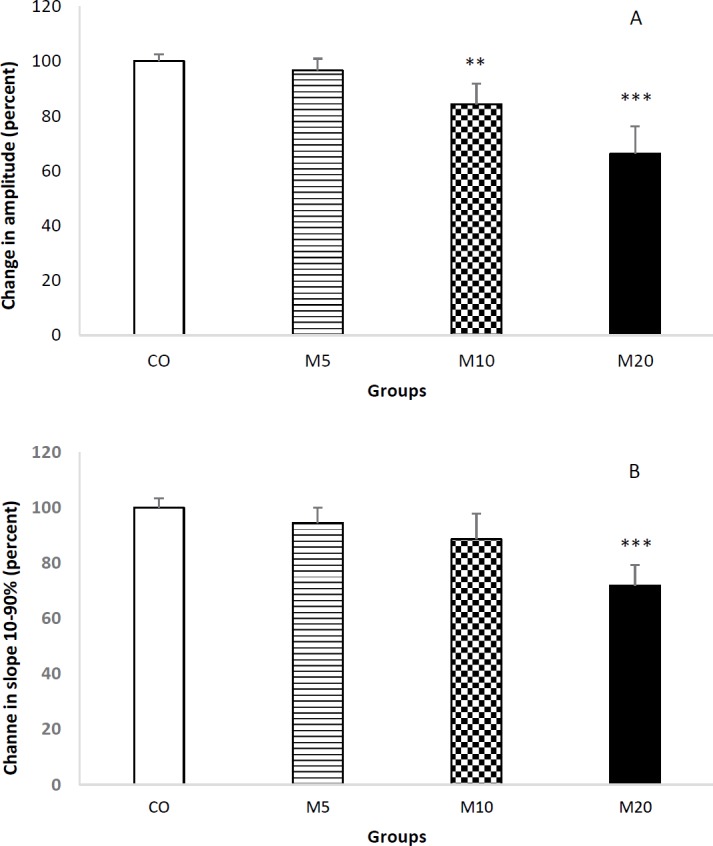
Comparison of amplitude (A) and slope 10-90% (B) of field excitatory post-synaptic potentials (fEPSPs) recorded from CA1 area of the rats. Receiving morphine during lactation period by the rats significantly decreased the amplitude and slope 10-90% of fEPSPs of their offspring. ** *P*<0.01 control group vs. M10 group; *** *P*<0.001 control group vs. M20 group

**Figure 5:**
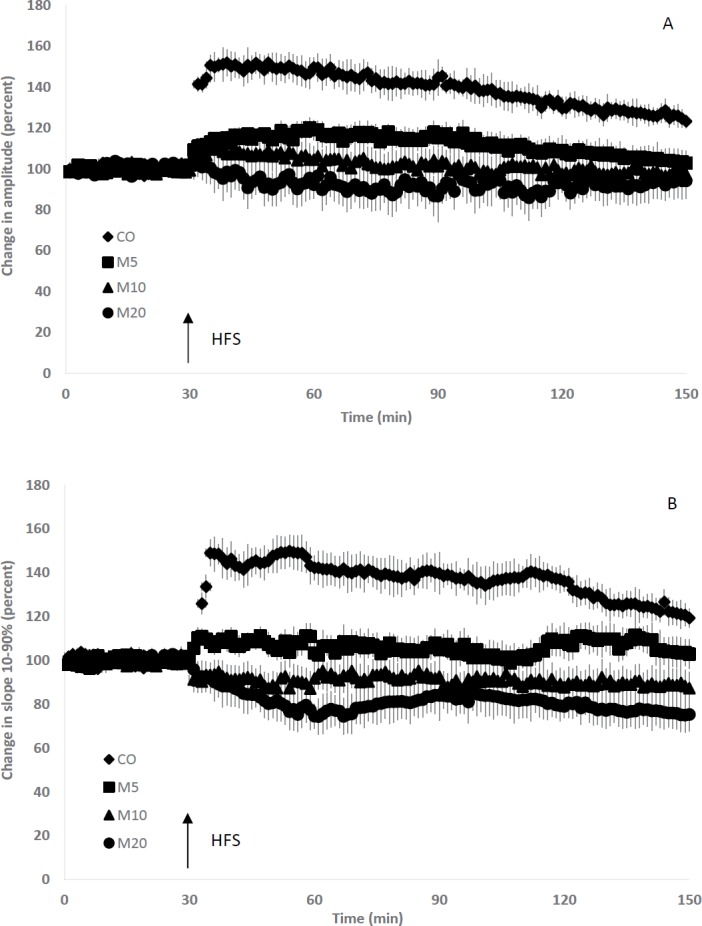
Induction of long term potentiation (LTP) in the field excitatory post-synaptic potentials (fEPSPs) recorded in the CA1 area after tetanization of the Schaffer's collaterals-CA1 pathway. Although high-frequency stimulation (HFS) elicited LTP in the control (CO) rats, it failed to potentiate responses in the other groups. There was statistically significant difference among the post-tetanus amplitude (A) and slope 10-90% (B) of fEPSPs of CO and the other groups (*P*<0.01 for all comparisons) and also between M5 and M20 rats (*P*<0.01)

***Spatial learning and memory***

On 45 days of postnatal age, when the brain is matured (22, 23), spatial learning and memory was investigated by Morris water maze (MWM) during four consecutive days with four trials per day (24). MWM was a black circular metal tank (150 cm in diameter and 70 cm in depth) that was filled with water (22 ^°^C) up to 20 cm below the rim. A circular platform (10 cm in diameter) was located 2 cm below the water surface in the center of one arbitrarily quadrant (northeast, southeast, southwest, and northwest). The animal’s navigation was monitored by a video camera that was connected to tracking software (Radiab 7.1, Iran). Each trial was started by releasing an animal into the maze facing the perimeter, at one of the four starting points (north, south, east, or west), which was randomly chosen by the software. The animals were permitted to swim for a maximum time of 60 sec to find the hidden platform. If the rat failed to find the platform, it was guided onto the platform manually. After 15 min, the rat was placed again in the pool from different starting point. The time elapsed and the distance traveled to find the hidden platform during each trial were measured as indices of the learning of the spatial task. After completing the learning trials, the probe trial test was also performed. The platform was removed from the pool and the rats were placed at the quadrant, which was opposite to the platform (target) quadrant and allowed to swim freely for 30 sec, and the time spent and the distance traveled in the target quadrant were recorded as spatial retention.

***In vivo electrophysiology***

As previously described (25), the rats were anesthetized with urethane (1.5 g/kg, IP) and placed in a stereotaxic apparatus (Borj Sanat, Iran). Two small holes (1 mm diameter) were drilled in the skull to allow the insertion of stimulating (4.2 mm posterior to bregma and 3.8 mm lateral to the midline) and recording (3.4 posterior to bregma and 205 mm lateral to the midline) electrodes in the brain. Electrodes were prepared from Teﬂon-coated stainless-steel wire (0.008 inch diameter, A-M Systems, USA) exposed only at the tip (tip separation 0.10 mm). A bipolar stimulating electrode was advanced through the Schaffer’s collaterals (2.8 mm below the skull) and a monopolar recording electrode was lowered to the CA1 stratum radiatum (2.5 mm below the skull). All coordinates were based on streotaxic atlas and adapted to the animals according to bregma-lambda distance. Proper location of the electrodes was determined using electrophysiological and stereotaxic indicators. Using computer software (eProbe, ScienceBeam, Iran), field excitatory post-synaptic potentials (fEPSPs) were recorded from the stratum radiatum of the CA1 area of the hippocampus in response to stimulation (two sweeps/min at 30 sec intervals) of the ipsilateral Schaffer’s collateral for 30 minutes. Then, using high-frequency stimulation (HFS) protocol (10 bursts of 10 stimuli, 0.2 msec stimulus duration, and 10 sec interburst intervals), LTP was induced. Following the LTP induction, recordings continued for at least 2 hr. The data were considered for the percent changes in amplitude and slope 10-90% of the pre- and post-tetanus responses.

***Statistical analysis***

The data are expressed as means±SEM. One-way analysis of variance (ANOVA) was performed on the Gellet–Holtzman score of different groups. The data of spatial learning and probe trial were analyzed with repeated measure ANOVA and one-way ANOVA, respectively. Two-way ANOVA was applied to the amplitude of fEPSPs. If the post-tetanus amplitude of fEPSPs was increased at least 20%, it was interpreted as LTP. Tukey’s test was applied as *post hoc*. All statistical analysis was performed with SPSS 20 software and *P*<0.05 were considered as significant difference.

## Results

***Withdrawal signs***

Evaluating the signs of withdrawal syndrome by the modified Gellert-Holtzman method revealed that receiving morphine during lactation period causes morphine-dependency of rats’ offspring, and they show withdrawal signs. Analysis of the overall Gellert-Holtzman score by ANOVA showed that there is statistically significant difference between the groups (F_3,36_=19.023; *P*<0.0001). The overall score of offspring of mothers who received 5 mg/kg morphine two times daily during lactation period was 16.11±5.36 (Figure 1) and increased to 27.46±7.94 and 31.49±4.51 in M10 and M20 groups, respectively. Tukey’s test showed that there is significant difference between CO and the other groups (*P*<0.001) and also between M5 group and M10 and M20 groups (*P*<0.01, for both comparison). 

***Spatial learning and memory***

The total time spent and the total distance traveled to find the hidden platform during each trial are considered as criteria of learning in the Morris water maze. Repeated ANOVA measurements demonstrated that the animals in all groups of the study learned the maze task during four days (F_3,584_=6.719; *P*<0.0001). As it is shown in Figure 2, the animals of all groups spent less time and traveled less distance to find the hidden platform during consecutive days of the study. Statistical analysis showed that there is a significant difference between mean time spent (*P*<0.0001) and mean distance traveled (*P*<0.0001) among all the groups. Also, results of Tukey’s test showed that there is significant difference between the CO group and M10 and M20 groups (*P*<0.05).

After finishing the last trial of learning phase, the hidden platform was removed from the maze and the rats were allowed to swim for 30 sec. If the animal remembers the location of hidden platform, it should typically spend longer time and travel more distance in the target quadrant. The statistics showed that there is significant difference among all groups for mean spent time (F_3,36_=4.412; *P*<0.0001) and mean traveled path (F_3,36_=6.913; *P*<0.0001) in the target quadrant. Analysis of the data indicated that morphine consumption during lactation period causes that the rats’ offspring spend less time and travel less distance in the target quadrant, dose-dependently. The results of Tukey’s test revealed that there is significand difference between the CO group and M10 (*P*<0.05) and M20 (*P*<0.01) groups for mean spent time (Figure 3A) and also between the CO and M20 groups (*P*<0.01) for mean traveled distance in the target quadrant (Figure 3B).

***In vivo electrophysiology***

Receiving morphine during lactation period decreased the amplitude and slope 10-90% of fEPSPs of rats’ offspring (two-way ANOVA, F_3,91_=6.736; *P*<0.0001). Compared to CO rats, the mean amplitude of fEPSPs of M5 rats decreased only 4 percent, but it decreased 16 and 34 percent in M10 and M20 animals, respectively. Results of Tukey’s *post hoc* revealed that there is significant difference between the mean amplitude of fEPSPs of CO and M10 (*P*<0.01) and also CO and M20 (*P*<0.001) groups (Figure 4). In addition, the mean slope 10-90% of fEPSPs of M20 group decreased about 30 percent compared to the CO group (*P*<0.001). 

After stimulating Schaffer’s collaterals by the HFS protocol, LTP was induced and fEPSPs were recorded for 2 hrs. LTP induction increased the mean amplitude and slope 10-90% of fEPSPs recorded from CA1 area of CO rats (up to 50 percent). The LTP lasted for about 2 hr (*P*<0.001 for comparison of before and after LTP induction). As it is shown in Figure 5, morphine consumption during lactation period inhibited LTP induction in the Schaffer’s collaterals-CA1 pathway of rats’ offspring. Although high-frequency stimulation of Schaffer’s collaterals of M5 rats increased the mean amplitude and slope 10-90% of fEPSPs (*P*<0.05), but did not reach the definite LTP level (at least 20% increase). Tukey’s results also revealed that there was no significant difference between pre- and post-tetanus amplitude and slope 10-90% of fEPSPs of M10 rats. In addition, the mean amplitude and slope 10-90% of fEPSPs recorded in CA1 area of M20 rats decreased about 15% and 20% after LTP induction, respectively (*P*<0.05 for both comparisons). There was statistically significant difference among the post-tetanus amplitude of fEPSPs of CO and the other groups (*P*<0.01 for all three comparisons) and also between M5 and M20 rats (*P*<0.01). Also, statistics revealed the differences among post-tetanus slope 10-90% of fEPSPs of CO and the treated groups (*P*<0.01 for all three comparisons) and between M5 and M20 rats (*P*<0.01).

## Discussion

Our results revealed that morphine consumption during lactation period causes morphine dependency of rats’ offspring. To our knowledge, there is no study for evaluating morphine in animals’ milk, but it has been shown that morphine is detected in human milk only 30 minutes after epidural injection (26). Likewise, results of another study showed that 0.8 to 12% of morphine received by the mother can be detected in serum of breastfed infant (27). It has been reported that morphine consumption during pregnancy and lactation causes dependency of 14 days old rat offspring and reduces antinociceptive effect of morphine (17). In addition, it has been shown that methadone consumption during pregnancy and lactation period increases sensitivity to nociceptive thresholds in rat offspring (28). 

We also demonstrated that morphine consumption during lactation period impairs the spatial learning and memory, decreases the mean amplitude and slope 10-90% of fEPSPs recorded from CA1 area of hippocampus, and inhibits LTP induction in these neural circuits of rats’ offspring. The proper structure and function of the hippocampal neural circuits is essential for many types of learning and memory, especially spatial learning and memory. It is not exactly clear how memories form, but two types of synaptic plasticity namely LTP and LTD have been accepted as key mechanisms of memory formation and have been well-studied in the hippocampus (4). LTP is long-lasting increase in signal transmission between two neurons, which is induced after high-frequency stimulation of the pre-synaptic neuron. By stimulating the circuit, glutamate releases from the pre-synaptic terminal and the post-synaptic α-amino-3-hydroxy-5-methyl-4-isoxazolepropionic acid (AMPA) receptors are then activated. AMPA receptors activation causes calcium entry to the post-synaptic terminal and makes an EPSP. HFS increases post-synaptic calcium concentration and causes the summation of EPSPs, which leads to LTP induction. Some types of LTP, especially the LTP in the Schaffer’s collateral to CA1 pathway is NMDA receptor-dependent (29). There are studies confirming that LTP induction in different area of hippocampus modulates spatial learning and memory (30).

The critical period of the brain development is a period of prenatal and early postnatal life in which the experiences of exposure to the environment in addition to genetic codes play an important role in the formation of synaptic connections (12). The hippocampus has a critical period of development like the other areas of the brain (13). It has been shown that morphine consumption during pregnancy and lactation period decreases the number of NMDA receptors in the brain of 14 days old rats’ offspring (18) and decreases the kinetics of these receptors in the hippocampus (20). Tao and colleagues have demonstrated that morphine consumption during pregnancy reduces the expression of NR1 and NR2B subunits of NMDA receptors in the CNS of rats’ offspring (31). In addition, it has been shown that morphine consumption during pregnancy decreases pyramidal neurons of different areas of the hippocampus of mice offspring (32). In line with these changes, researchers have revealed that prenatal morphine exposure impairs passive avoidance learning and memory of rats, which is probably due to decrease in brain-derived neurotrophic factor (BDNF) expression in their hippocampus (33). 

Besides these studies, there are several studies indicating that morphine can impair learning and memory processes (34) via μ opioid receptors (35). Yang *et al*. have demonstrated that impairment of spatial learning of prenatally morphine-exposed rats is due to decreasing of Serine 133-phosphorylated CREB, which has an essential role in learning process (16).

## Conclusion

Morphine consumption during lactation period impairs spatial learning and memory and also decreases synaptic plasticity of hippocampal neural circuits of rat offspring. These may be due to changes in the structure of hippocampal neural circuits and function of neurotransmitters and receptors in the hippocampus. 
